# Accuracy of Non-Exercise Estimated Cardiorespiratory Fitness in Japanese Adults

**DOI:** 10.3390/ijerph182312288

**Published:** 2021-11-23

**Authors:** Robert A. Sloan, Marco V. Scarzanella, Yuko Gando, Susumu S. Sawada

**Affiliations:** 1 Department of Social and Behavioral Medicine, Kagoshima University, Kagoshima 890-8520, Japan; 2 Department of Science and Engineering, Kagoshima University, Kagoshima 890-0065, Japan; marco.visentiniscarzanella@gmail.com; 3 Department of Sport Science, Surugadai University, Hannō 357-8555, Japan; gando.yuko@surugadai.ac.jp; 4 Faculty of Sport Sciences, Waseda University, Tokorozawa-shi 359-1192, Japan; yususumi@gmail.com

**Keywords:** estimated cardiorespiratory fitness, electronic medical records, epidemiology, public health

## Abstract

Cardiorespiratory fitness (CRF) is an independent predictor of morbidity and mortality. In Japan, annual physical exams are mandatory in workplace settings, and most healthcare settings have electronic medical records (EMRs). However, in both settings, CRF is not usually determined, thereby limiting the potential for epidemiological investigations using EMR data. PURPOSE: To estimate CRF (mL/kg/min) using variables commonly recorded in EMRs. METHODS: Participants were 5293 Japanese adults (11.7% women) who completed an annual physical exam at a large gas company in Tokyo, Japan, in 2004. The mean age was 48.3 ± 8.0 years. Estimated CRF (eCRF) was based on age, measured body mass index, resting heart rate, systolic and diastolic blood pressure, and smoking. Measured CRF was determined by a submaximal cycle ergometer graded exercise test. RESULTS: Regression models were used for males and females to calculate Pearson’s correlation and regression coefficients. Cross-classification of measured CRF and eCRF was conducted using the lowest quintile, quartile, and tertile as the unfit categories. R’s for eCRF were 0.61 (MD 4.41) for men and 0.64 (MD 4.22) for women. The overall accuracy level was reasonable and consistent across models, yet the unfit lower tertile model provided the best overall model when considering the positive predictive value and sensitivity. CONCLUSION: eCRF may provide a useful method for conducting investigations using data derived from EMRs or datasets devoid of CRF or physical activity measures.

## 1. Introduction

Cardiorespiratory fitness (CRF) is a health-related component of physical fitness driven by the ability of the cardiovascular, musculoskeletal, and respiratory systems to utilize oxygen during bouts of physical activity [1]. A large body of evidence has identified low CRF (unfit) as a predictor for heart disease, diabetes, stroke, cancer, hypertension, depression, poor sleep, and falls [2]. A recent meta-analysis of 102,980 participants showed that unfit adults have a 70% increase in the risk of premature death and a 56% increase in the risk of heart disease than those who are more fit [3]. In epidemiological investigations, being unfit has been categorized as the lowest quintile, quartile, or tertile of CRF [2]. Given the strength of evidence, physicians and researchers have recently called for CRF to be considered a clinical vital sign that is best measured by a graded exercise test [2]. Because graded exercise testing can be impractical due to cost, equipment, time, and staffing, researchers have developed non-exercise fitness testing models to estimate CRF (eCRF) [2,4].

Most eCRF models have used some combination of CRF correlates, such as age, body mass index (BMI), resting heart rate, smoking status, and physical activity status (PAS) [2,4]. Compared to measured CRF, eCRF models with PAS have demonstrated moderate (R = 0.62) to high (R = 0.86) correlations and high (90%) accuracy to classify unfitness along with the ability to predict mortality and morbidity on a par with measured CRF [5,6]. Given the type of variables used to calculate eCRF, a potentially rich data source may be found in electronic medical records (EMRs). However, EMRs do not typically have PAS information; therefore, it is impossible to conduct epidemiological studies using existing EMR data using PAS-based eCRF models [7]. However, EMRs do contain independent variables that have been used in previous eCRF PAS models (age, gender, BMI, resting heart rate, blood pressure, smoking) [8]. Therefore, it may be possible to create nuanced equations to calculate eCRF without PAS.

Currently, there are a few peer-reviewed journal investigations that have developed eCRF models in populations without PAS by simply using gender, age, and BMI [4,9,10,11]. The usefulness of such equations has been questioned due to the array of correlation values with no or poor accuracy reported [4,9,11,12]. In a recent review article, Wang et al. stated that eCRF equations without PAS measurement require further investigation across different populations and would be broadly useful if validated [4].

To date, only one eCRF study without PAS has been conducted in an Asian population and was found to have very low accuracy for classifying unfit individuals [10,11]. Because Asian populations are physiologically different from Western populations and generally have lower CRF levels for a given age and body mass index, new eCRF equations without PAS may be warranted [4,12,13,14]. A well-established Japanese cohort with measured CRF can develop eCRF models without PAS [15]. The cohort was established in 1985, tracking more than 10,000 working male and female adults. Several fundamental cohort studies have been published on the prospective relationship between objectively measured low CRF and diabetes incidence, cancer mortality, and hypertension incidence [16,17,18]. One limitation of the Japanese cohort studies has been the lack of prospective studies on females. Given the voluminous data in EMRs, eCRF equations without PAS that identify unfit females may help address gaps in the literature. For example, it would be possible to aggregate data from different hospitals over the past 20 years and determine breast cancer incidence using eCRF without PAS as an independent variable.

Therefore, the primary aim of this study was to develop new models for eCRF without PAS that could potentially be used in large-scale investigations using EMR data. Our hypothesis was that eCRF models without PAS can reasonably identify unfit adults.

## 2. Methods

Data were obtained on Japanese adults (N = 5596) who completed an annual health check at a large gas company in the Tokyo area in Japan in 2004. The mean age was 48.5 ± 7.9 years for men and 46.3 ± 8.3 years women. Exclusion criteria consisted of those with abnormal electrocardiograms; histories of myocardial infarctions, diabetes, strokes, or cancers; BMI < 18.5 kg/m^2^; or failure to achieve at least 85% of maximal heart rate (220 minus age in years) during a graded exercise test. Those with missing data pertinent to this investigation were also excluded. In all, 303 participants were excluded, providing a final N = 5293 (11.7% women). All individuals completed informed consent, and the National Institutes of Biomedical Innovation, Health, and Nutrition (NIBIOHN) research ethics committee approved the current study’s methodology, protocol, and procedures. The health examinations were performed under Japanese Industrial Safety and Health Law. All information from the health examinations was used only in aggregate without reference to or disclosing individual information. For the current study, a de-identified limited dataset was collected, used, and approved by the NIBIOHN. A detailed description of the methods and protocols used in this investigation is already published, and new additions or variations have been stated [16,17].

## 3. Measurements

### Clinical Examination

Predictor variables were assessed during an annual health examination that included objective measurements of age (Age), BMI, resting heart rate (rHR), systolic blood pressure (SBP), diastolic blood pressure (DBP), and self-reported smoking status (yes/no) in 2004. Age was verified from each individual’s employment record. Height (Ht) and weight (Wt) were measured on a calibrated scale in centimeters and kilograms, respectively. An automated sphygmomanometer measured the resting blood pressure and heart rate with the participant in a sitting position. American College of Sports Medicine Guidelines were used to conduct VO_2_max (mL/kg/min) testing and determine CRF for each participant by the Åstrand–Rhyming cycle ergometer test [1]. Heart rate was measured using the RR interval on an electrocardiogram. Target heart rate was set as 85% of maximum heart rate estimated from age (220—age), and load (kg) was gradually added per the Åstrand–Rhyming protocol [1]. VO2max was calculated using the Åstrand–Rhyming nomogram, based on the peak heart rate obtained from the last 1 min of each participant’s peak load and the Åstrand age correction factors [1]. This method of determining VO_2_max has been shown to strongly correlate (r = 0.92) with results determined using the gold standard method [19,20].

## 4. Analysis

### 4.1. Regression

Using a machine learning supervised learning technique, we conducted separate linear regression analyses for males and females to predict eCRF based on nonlinear augmentation of the predictor variables [21]. The data were first examined for outliers and skewness. We removed the outliers from the dataset by removing participants with more continuous variables beyond the +3 σ interval. After removing outliers, the dataset comprised 4675 males and 618 females, 94.6% of the original participants. The male and female eCRF prediction equations were formulated to minimize the average Mean Squared Error (MSE), where N is the number of samples in our dataset and $MSE=\frac{1}{N}\sum_{i=1}^{N}{\left(\hat{y_{i}}-y_{i}\right)}^{2}$. The prediction equation used Age, Ht, Wt, BMI, rHR, SBP, DBP, and smoking. All variables were continuous except for smoking (smoking = 1, nonsmoker = 0). The data were standardized by subtracting the mean and dividing by the standard deviation for each variable. Next, separate models were then trained for male and female participants.

We augmented the original eight variables with second-order and interaction terms and regressed them linearly to the dependent variable for training. In this way, the nonlinearity was transferred from the regressor to the independent variables, while the model’s overall interpretability was maintained. The augmentation procedure added the following 28-s order and interaction terms: (Wt^2^, Wt × Ht, Wt × Age, Wt × rHR, Wt × SBP, Wt × DBP, Wt × BMI, Ht^2^, Ht × Age, Ht × rHR, Ht × SBP, Ht × DBP, Ht × BMI, Age^2^, Age × rHR, Age × SBP, Age × DBP, Age × BMI, rHR^2^, Ht × SBP, Ht × DBP, rHR × BMI, SBP^2^, SBP × DBP, SBP × BMI, DBP^2^, and DBP × BMI), for a total of 36 variables. Because smoking was a categorical variable, it was not used to create the additional variables. The augmented dataset was inputted into an elastic net linear regressor [8] and trained and evaluated through 10-fold cross-validation. Optimal model hyperparameters were found to be (α = 0.022, λ =1.0) for the male and (α = 0.001, λ = 0.5) for the female datasets through the cross-validation procedure. R was then calculated using the eCRF equations for male and female datasets in Supplementary Material S1. Lastly, we also cross-validated a well-documented Western eCRF equation without PAS (77.96 − 10.35 (sex; M = 0, F = 1) − 0.92 (BMI) − 0.32 (age)) to see how it would perform [9].

### 4.2. Fitness Classification

After determining correlations, we used the same dataset in a classification scenario, testing how well a given participant could be categorized as unfit or fit. This cross-classification of measured CRF and eCRF was conducted using established epidemiological thresholds to classify unfit individuals (lowest quintile, lowest quartile, and lowest tertile) [2]. The equation, eCRF>α, where α served as the value of the α^th^ percentile in the CRF distribution grouped by gender, was used. Once classified, we determined the accuracy (ACC), sensitivity (SEN), and positive predictive values (PPV) of eCRF to detect unfit individuals compared to measured CRF for each threshold. Being ‘positive’ was equivalent to being in the ‘unfit’ category. All analyses were performed in scikit-learn version 0.22.2 (O’reilly, Sebastopol, CA, USA).

## 5. Results

Descriptive statistics of the study population are presented in Table 1. Correlation coefficients between each independent variable and CRF are presented in Supplementary Material S1. The correlations and mean deviations (mL/kg/min) for eCRF were R = 0.61 (MD 4.41) for men and R = 0.64 (MD 4.22) for women. The models explained 37% (SEE, 95% CI; 5.64, 0.15–13.33) and 40% (SEE, 95% CI; 5.62, 0.17–14.09) variance in CRF for men and women, respectively. We also tested advanced machine learning models (gradient-boosted trees and neural networks). The models were not superior to the linear model and did not provide visible algorithms (i.e., black box).

Supplementary Material S1 provides the detailed men’s and women’s eCRF equations and a publicly accessible Google Sheet for practical application and reproducibility. Comparatively, when we cross-validated the eCRF Western equation with our dataset, we found R = 0.36 (MD 6.61) for males and R = 0.14 (MD 6.65) for females. Table 2 provides the findings regarding the ACC, PPV, and SEN values using the lowest quintile, quartile, and tertile to classify being unfit for males and females, respectively. While accuracy levels were consistent (~72% for males and ~75% females), the unfit lower tertile model provided the best overall model when considering positive predictive values and sensitivity. Based on a residual plot (Supplementary Material S1), we found the model was most accurate for VO_2_max values in the 30–40 mL/kg/min range but tended to underestimate higher VO_2_max values > 40 mL/kg/min.

## 6. Discussion

This study aimed to compare eCRF with a reference measure of CRF in a large Japanese population. Using clinical measures typically captured in EMRs, we found eCRF models without PAS to provide moderate correlations and accuracy compared to reference CRF measures. The moderate predictive values are useful given eCRF is a no-cost and non-diagnostic test [22]. This is the first study to report the association of objectively measured CRF with eCRF without PAS in a Japanese cohort to the best of our knowledge. Overall, our findings show that eCRF without PAS may serve as a useful marker for fitness for epidemiological investigations.

Although this is the first study of its kind, Cao et al. conducted investigations in Japanese males (*n* = 148) and females (*n* = 127) and developed eCRF PAS-based equations using age, BMI, measured steps per day, and minutes of vigorous physical activity [23,24]. The authors found strong correlations, R = 0.85 (SEE 3.11) and R = 0.84 (SEE 4.15), compared to maximal cycle ergometer testing [23,24]. More recently, Matsuo et al. developed an eCRF PAS-based equation compared to maximal treadmill test in working Japanese adults (*n* = 198) and found a strong correlation (*r* = 0.77) [25]. As expected, the PAS model correlations are higher than our eCRF model without PAS; beyond genetics, PAS is an essential variable for modifying CRF [2,4,11]. Though these eCRF PAS-based correlations are higher, access to specific PAS data from existing EMR datasets is not likely to have been recorded in EMRs [7,25].

Some equations developed without PAS predict CRF using age, BMI, and gender [4,11,12]. According to these simple equations, individuals of the same age, height, and weight would have the same CRF level [26,27]. Jang et al. developed an eCRF model without PAS and reported a high correlation (R = 0.82) in working-age Korean adults (*n* = 217). We cross-validated a similar equation from Baynard et al. and found weak correlations with CRF [9]. These simplified models may be somewhat limited, because it is unlikely that a large group of individuals of the same BMI and age would have the same fitness level given that CRF is influenced by multiple biological and lifestyle factors [2,28,29,30]. Moreover, despite the high correlations reported, Peterman et al. recently found Baynard’s and Jang’s equations to have poor accuracy for classifying unfit (lowest tertile) individuals at 37% and 2%, respectively [11,27]. Poor accuracy in determining unfit individuals may limit the ability to conduct epidemiological investigations [2,5,11,12].

### Limitations

This study is not without limitations. Our study’s primary limitation regarding correlation was that the referent CRF was conducted using a submaximal test to determine VO_2_max. Although highly correlated (r = 0.92) with maximal exercise testing, submaximal testing has overestimated VO_2_max in older adults and underestimated VO_2_max in trained adults, partly due to general maximal heart rate estimation equations being used [2,20]. Despite this limitation, our classification analyses demonstrated that eCRF is a useful tool to identify unfit individuals. The generalizability of the findings may be limited because participants were working Japanese adults 18–64 years old. On the other hand, the sample’s homogeneity strengthened the internal validity of our results by limiting possible physiological and socioeconomic confounders and may apply to a large segment of the Japanese population and other East Asian cultures [31].

## 7. Conclusions

In summary, eCRF without PAS may be a practical tool for identifying unfit Japanese adults and conducting EMR data-driven epidemiological investigations. Future research should focus on conducting validation studies of eCRF as a predictor of health outcomes and determine how well eCRF can detect changes in fitness over time.

## Figures and Tables

**Table 1:** Baseline characteristics of participants.

	All (*n* = 5293)	Males (*n* = 4675)	Females (*n* = 618)
Age, year	48.3 (8.0)	48.5 (7.9)	46.3 (8.3)
BMI, kg/m^2^	23.3 (2.8)	23.6 (2.7)	20.9 (2.5)
Resting Heart Rate, bpm	71.0 (9.8)	70.6 (9.8)	73.6 (9.5)
Systolic Blood Pressure, mmHg	123.8 (17.0)	125.0 (16.7)	115.2 (16.3)
Diastolic Blood Pressure, mmHg	77.6 (11.0)	78.5 (10.7)	70.7 (10.1)
Smoker, %	3181 (60.1%)	3112 (66.6%)	69 (11.2%)
Estimated CRF, mL/kg/min	38.7 (4.4)	39.0 (4.3)	36.3 (4.6)
Measured CRF, mL/kg/min	38.7 (7.2)	39.0 (7.1)	36.3 (7.3)

Data represent mean (standard deviation) or number (percentages). BMI: body mass index; bpm: beats per minute; CRF: cardiorespiratory fitness (maximal oxygen consumption).

**Table 2:** Accuracy of estimating CRF according to measured CRF.

Group	Unfit (Lowest Quintile)			Unfit (Lowest Quartile)			Unfit (Lowest Tertile)		
	ACC	PPV	SEN	ACC	PPV	SEN	ACC	PPV	SEN
Male	72.4%	36.3%	73.3%	71.9%	44.0%	74.5%	71.5%	50.9%	73.3%
Female	74.9%	35.2%	72.9%	74.8%	43.3%	73.2%	76.4%	59.9%	74.7%

ACC: accuracy, PPV: positive predictive value, SEN: sensitivity. eCRF means (SD) for the male quintile, quartile, and tertile groups were 35.3 (3.2), 35.7 (3.3), and 36.0 (3.3) mL/kg/min, respectively. eCRF means (SD) for the female quintile, quartile, and tertile groups were 31.8 (3.2), 32.2 (3.3), and 32.8 (3.2) mL/kg/min, respectively.

## Data Availability

The data are not publicly available and are maintained by NIBIOHN.

## Supplementary Materials

The following are available online at https://www.mdpi.com/article/10.3390/ijerph182312288/s1. S1: Correlations between independent variables, equations, Google Sheet link, and residual plot.

## References

[1] American College of Sports Medicine A. (2017). ACSM’s Guidelines for Exercise Testing and Prescription.

[2] Ross R., Blair S.N., Arena R., Church T.S., Després J.-P., Franklin B.A., Haskell W.L., Kaminsky L.A., Levine B.D., Lavie C.J. (2016). Importance of Assessing Cardiorespiratory Fitness in Clinical Practice: A Case for Fitness as a Clinical Vital Sign: A Scientific Statement from the American Heart Association. Circulation.

[3] Kodama S., Saito K., Tanaka S., Maki M., Yachi Y., Asumi M., Sugawara A., Totsuka K., Shimano H., Ohashi Y. (2009). Cardiorespiratory Fitness as a Quantitative Predictor of All-Cause Mortality and Cardiovascular Events in Healthy Men and Women: A meta-analysis. JAMA.

[4] Wang Y., Chen S., Lavie C.J., Zhang J., Sui X. (2019). An Overview of Non-exercise Estimated Cardiorespiratory Fitness: Estimation Equations, Cross-Validation and Application. J. Sci. Sport Exerc..

[5] Artero E.G., Jackson A.S., Sui X., Lee D.-C., O’Connor D.P., Lavie C.J., Church T.S., Blair S.N. (2014). Longitudinal Algorithms to Estimate Cardiorespiratory Fitness: Associations with nonfatal cardiovascular disease and disease-specific mortality. J. Am. Coll. Cardiol..

[6] Stamatakis E., Hamer M., O’Donovan G., Batty G.D., Kivimaki M. (2012). A non-exercise testing method for estimating cardiorespiratory fitness: Associations with all-cause and cardiovascular mortality in a pooled analysis of eight population-based cohorts. Eur. Heart J..

[7] Lindeman C., McCurdy A., Lamboglia C.G., Wohlers B., Pham A.N.Q., Sivak A., Spence J.C. (2020). The extent to which family physicians record their patients’ exercise in medical records: A scoping review. BMJ Open.

[8] Jackson A.S., Sui X., O’Connor D.P., Church T.S., Lee D.-C., Artero E.G., Blair S.N. (2012). Longitudinal Cardiorespiratory Fitness Algorithms for Clinical Settings. Am. J. Prev. Med..

[9] Baynard T., Arena R.A., Myers J., Kaminsky L.A. (2016). The Role of Body Habitus in Predicting Cardiorespiratory Fitness: The FRIEND Registry. Int. J. Sports Med..

[10] Jang T.-W., Park S.-G., Kim H.-R., Kim J.-M., Hong Y.-S., Kim B.-G. (2012). Estimation of Maximal Oxygen Uptake without Exercise Testing in Korean Healthy Adult Workers. Tohoku J. Exp. Med..

[11] Peterman J.E., Whaley M.H., Harber M.P., Fleenor B.S., Imboden M.T., Myers J., Arena R., Kaminsky L.A. (2021). Comparison of non-exercise cardiorespiratory fitness prediction equations in apparently healthy adults. Eur. J. Prev. Cardiol..

[12] Hsieh S.D., Yoshinaga H., Muto T. (2003). Waist-to-height ratio, a simple and practical index for assessing central fat distribution and metabolic risk in Japanese men and women. Int. J. Obes..

[13] Ghouri N., Purves D., McConnachie A., Wilson J., Gill J.M.R., Sattar N. (2013). Lower cardiorespiratory fitness contributes to increased insulin resistance and fasting glycaemia in middle-aged South Asian compared with European men living in the UK. Diabetologia.

[14] Iliodromiti S., Ghouri N., Celis-Morales C.A., Sattar N., Lumsden M.A., Gill J.M.R. (2016). Should Physical Activity Recommendations for South Asian Adults Be Ethnicity-Specific? Evidence from a Cross-Sectional Study of South Asian and White European Men and Women. PLoS ONE.

[15] Sloan R.A., Sawada S.S., Lee I.M., Gando Y., Kawakami R., Okamoto T., Tsukamoto K., Miyachi M. (2018). The Association of Fit-Fat Index with Incident Diabetes in Japanese Men: A Prospective Cohort Study. Sci. Rep..

[16] Sawada S.S., Muto T., Tanaka H., Lee I.M., Paffenbarger R.S., Shindo M., Blair S.N. (2003). Cardiorespiratory fitness and cancer mortality in Japanese men: A prospective study. Med. Sci. Sports Exerc..

[17] Sawada S.S., Lee I.M., Muto T., Matuszaki K., Blair S.N. (2003). Cardiorespiratory fitness and the incidence of type 2 diabetes: Prospective study of Japanese men. Diabetes Care.

[18] Momma H., Sawada S.S., Sloan R.A., Gando Y., Kawakami R., Miyachi M., Fukunaka Y., Okamoto T., Tsukamoto K., Nagatomi R. (2019). Frequency of achieving a ‘fit’ cardiorespiratory fitness level and hypertension: A cohort study. J. Hypertens..

[19] Cink R.E., Thomas T.R. (1981). Validity of the Astrand-Ryhming nomogram for predicting maximal oxygen intake. Br. J. Sports Med..

[20] Teraslinna P., Ismail A.H., MacLeod D.F. (1966). Nomogram by Astrand and Ryhming as a predictor of maximum oxygen intake. J Appl. Physiol..

[21] Wiemken T.L., Kelley R.R. (2020). Machine Learning in Epidemiology and Health Outcomes Research. Annu. Rev. Public Health.

[22] Trevethan R. (2017). Sensitivity, Specificity, and Predictive Values: Foundations, Pliabilities, and Pitfalls in Research and Practice. Front. Public Health.

[23] Cao Z.B., Miyatake N., Higuchi M., Miyachi M., Ishikawa-Takata K., Tabata I. (2010). Predicting VO2max with an objectively measured physical activity in Japanese women. Med. Sci. Sports Exerc..

[24] Cao Z.-B., Miyatake N., Higuchi M., Miyachi M., Ishikawa-Takata K., Tabata I. (2010). Predicting VO2max with an objectively measured physical activity in Japanese men. Eur. J. Appl. Physiol..

[25] Matsuo T., So R., Takahashi M. (2020). Workers’ physical activity data contribute to estimating maximal oxygen consumption: A questionnaire study to concurrently assess workers’ sedentary behavior and cardiorespiratory fitness. BMC Public Health.

[26] Chow L.S., Odegaard A.O., Bosch T.A., Bantle A.E., Wang Q., Hughes J., Carnethon M., Ingram K.H., Durant N., Lewis C.E. (2016). Twenty year fitness trends in young adults and incidence of prediabetes and diabetes: The CARDIA study. Diabetologia.

[27] Peterman J.E., Harber M.P., Imboden M.T., Whaley M.H., Fleenor B.S., Myers J., Arena R., Kaminsky L.A. (2021). Accuracy of Exercise-based Equations for Estimating Cardiorespiratory Fitness. Med. Sci. Sports Exerc..

[28] Committee PAGA (2018). 2018 Physical Activity Guidelines Advisory Committee Scientific Report.

[29] Shikany J.M., Jacobs D.R., Lewis C.E., Steffen L.M., Sternfeld B., Carnethon M.R., Richman J.S. (2013). Associations between food groups, dietary patterns, and cardiorespiratory fitness in the Coronary Artery Risk Development in Young Adults study. Am. J. Clin. Nutr..

[30] Santos R., Mota J., Okely A.D., Pratt M., Moreira C., Coelho-e-Silva M.J., Vale S., Sardinha L.B. (2014). The independent associations of sedentary behaviour and physical activity on cardiorespiratory fitness. Br. J. Sports Med..

[31] Wang Y., Lu D., Chung Y.J., Xu S. (2018). Genetic structure, divergence and admixture of Han Chinese, Japanese and Korean populations. Hereditas.

