# Supplementary material for: Accuracy of Non-Exercise Estimated Cardiorespiratory Fitness in Japanese Adults
Source: Int J Environ Res Public Health. 2021 Nov 23;18(23):12288. doi: 10.3390/ijerph182312288 (PMC8656653; doi:10.3390/ijerph182312288)
Supplement: Supplementary file 1 [file ijerph-18-12288-s001.zip › Supplement S1.pdf]

Accuracy of Non-exercise Estimated Cardiorespiratory Fitness in Japanese Adults

Supplement S1

Correlations between independent variables and CRF

**Male**		**Female**	
Variable	Pearson's correlation coefficient	Variable	Pearson's correlation coefficient
**Age**	**-0.29**	**Age**	**-0.24**
**SBP**	**-0.29**	**SBP**	**-0.31**
**DBP**	**-0.30**	**DBP**	**-0.29**
**Resting Heart Rate**	**-0.38**	**Resting Heart Rate**	**-0.38**
**BMI**	**-0.34**	**BMI**	**-0.43**
**Smoker**	**0.08**	**Smoker**	**0.07**

Accuracy of Non-exercise Estimated Cardiorespiratory Fitness in Japanese Adults

Male eCRF equation

$$
\begin{aligned}
eCRF = & -1.92\frac{Age - 48.51}{7.87} - 1.69\frac{Wt - 68.02}{8.79} - 2.68\frac{rHR - 70.61}{9.75} - 0.82\frac{BMI - 23.58}{2.65} \\
& - 0.36\frac{SBP - 124.96}{16.73} + 0.30\left(\frac{Age - 48.51}{7.87}\right)^2 \\
& + 0.02\left(\frac{Age - 48.51}{7.87}\right)\left(\frac{Ht - 169.76}{5.61}\right) - 0.38\left(\frac{Age - 48.51}{7.87}\right)\left(\frac{rHR - 70.61}{9.75}\right) \\
& - 0.01\left(\frac{Age - 48.51}{7.87}\right)\left(\frac{BMI - 23.59}{2.65}\right) + 0.09\left(\frac{Wt - 69.02}{8.79}\right)^2 \\
& + 0.14\left(\frac{Wt - 69.02}{8.79}\right)\left(\frac{DBP - 78.54}{10.73}\right) - 0.14\left(\frac{Ht - 169.76}{5.61}\right)\left(\frac{rHR - 70.61}{9.75}\right) \\
& + 0.09\left(\frac{Ht - 169.76}{5.61}\right)\left(\frac{BMI - 23.59}{2.65}\right) + 0.29\left(\frac{rHR - 70.61}{9.75}\right)^2 \\
& + 0.34\left(\frac{rHR - 70.61}{9.75}\right)\left(\frac{BMI - 23.59}{2.65}\right) - 0.06\left(\frac{rHR - 70.61}{9.75}\right)\left(\frac{SBP - 124.96}{16.73}\right) \\
& - 0.07\left(\frac{BMI - 23.59}{2.65}\right)\left(\frac{SBP - 124.96}{16.73}\right) + 0.27\left(\frac{SBP - 124.96}{16.73}\right)^2 \\
& - 0.04\left(\frac{DBP - 78.54}{10.73}\right) + 0.74(1 - Smoking) + 37.78
\end{aligned}
$$

Female eCRF equation

$$
\begin{aligned}
eCRF = & -1.08\frac{Age - 46.29}{8.29} - 1.53\frac{Wt - 51.90}{6.63} - 2.51\frac{rHR - 73.60}{9.45} - 1.57\frac{BMI - 20.94}{2.54} \\
& - 1.56\frac{SBP - 115.22}{16.26} + 1.08\frac{DBP - 70.71}{10.09} + 0.36\left(\frac{Age - 46.29}{8.29}\right)^2 \\
& - 0.46\left(\frac{Age - 46.29}{8.29}\right)\left(\frac{Wt - 51.90}{6.63}\right) + 0.49\left(\frac{Age - 46.29}{8.29}\right)\left(\frac{Ht - 157.44}{5.17}\right) \\
& - 0.31\left(\frac{Age - 46.29}{8.29}\right)\left(\frac{rHR - 73.60}{9.45}\right) - 0.48\left(\frac{Age - 46.29}{8.29}\right)\left(\frac{SBP - 115.22}{16.26}\right) \\
& + 0.78\left(\frac{Age - 46.29}{8.29}\right)\left(\frac{DBP - 70.71}{10.09}\right) - 1.06\left(\frac{Wt - 51.90}{6.63}\right)^2 \\
& + 1.39\left(\frac{Wt - 51.90}{6.63}\right)\left(\frac{Ht - 157.44}{5.17}\right) + 0.20\left(\frac{Wt - 51.90}{6.63}\right)\left(\frac{rHR - 73.60}{9.45}\right) \\
& - 1.76\left(\frac{Wt - 51.90}{6.63}\right)\left(\frac{SBP - 115.22}{16.26}\right) - 0.24\left(\frac{Wt - 51.90}{6.63}\right)\left(\frac{DBP - 70.71}{10.09}\right) \\
& - 0.04\left(\frac{Ht - 157.44}{5.17}\right)\left(\frac{rHR - 73.60}{9.45}\right) + 0.60\left(\frac{Ht - 157.44}{5.17}\right)\left(\frac{BMI - 20.94}{2.54}\right) \\
& + 0.54\left(\frac{Ht - 157.44}{5.17}\right)\left(\frac{SBP - 115.22}{16.26}\right) + 0.50\left(\frac{Ht - 157.44}{5.17}\right)\left(\frac{DBP - 70.71}{10.09}\right) \\
& + 0.09\left(\frac{rHR - 73.60}{9.45}\right)^2 + 0.55\left(\frac{rHR - 73.60}{9.45}\right)\left(\frac{BMI - 20.94}{2.54}\right) \\
& - 0.45\left(\frac{rHR - 73.60}{9.45}\right)\left(\frac{SBP - 115.22}{16.26}\right) + 0.41\left(\frac{rHR - 73.60}{9.45}\right)\left(\frac{DBP - 70.71}{10.09}\right) \\
& + 1.30\left(\frac{BMI - 20.94}{2.54}\right)^2 + 1.24\left(\frac{BMI - 20.94}{2.54}\right)\left(\frac{SBP - 115.22}{16.26}\right) \\
& + 0.57\left(\frac{BMI - 20.94}{2.54}\right)\left(\frac{DBP - 70.71}{10.09}\right) + 0.85\left(\frac{SBP - 115.22}{16.26}\right)^2 \\
& - 0.85\left(\frac{SBP - 115.22}{16.26}\right)\left(\frac{DBP - 70.71}{10.09}\right) + 0.17\left(\frac{DBP - 70.71}{10.09}\right)^2 - 1.71 Smoking \\
& + 36.51
\end{aligned}
$$

Google sheet link:
https://docs.google.com/spreadsheets/d/1lnYl4u62AMSUxkzyzKsKBHe8vr3WzLVRYxGyYE7xoGQ/edit#gid=1558519083

Accuracy of Non-exercise Estimated Cardiorespiratory Fitness in Japanese Adults

Residual plot

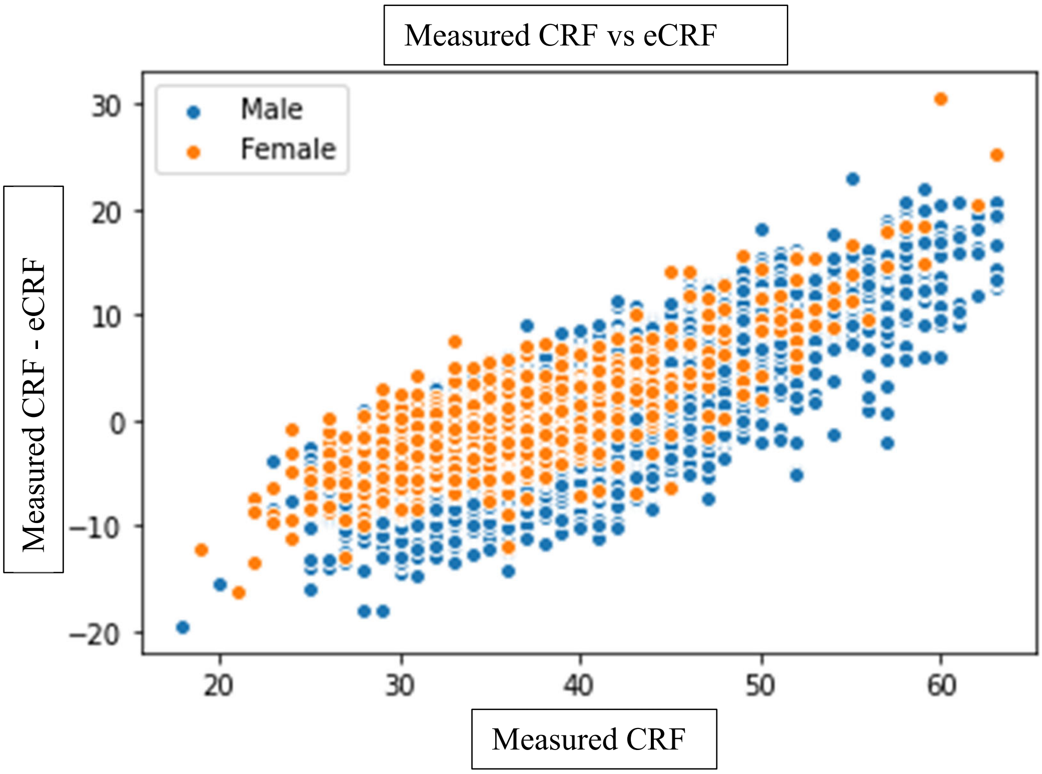

The model was most accurate when eCRF was in the 30 − 40 mL/kg/min range.
